# Immunotherapy-Based Combination Therapy for the Successful Treatment of Coexisting Primary and Axillary Accessory Breast Cancers: A Case Report

**DOI:** 10.7759/cureus.83340

**Published:** 2025-05-02

**Authors:** Yuko Tanaka, Yoshio Suzuki

**Affiliations:** 1 Cancer Genome, Dokkyo Medical University Hospital, Mibu, JPN; 2 Breast Center, Asahi General Hospital, Asahi, JPN; 3 Clinical Pathology, Asahi General Hospital, Asahi, JPN

**Keywords:** accessory breast cancer, cancer genome profiling, double cancers, immune-combined therapy, programmed death ligand 1 (pd-l1)

## Abstract

Accessory breast cancer is a relatively rare condition, and to our knowledge, no previous reports have described synchronous ipsilateral cancers of the breast and the axillary accessory breast. We report a unique case involving a 70-year-old female patient with luminal A subtype breast cancer and triple-negative accessory breast cancer. The axillary tumor, which had become ulcerative and exudative, was diagnosed as an accessory breast cancer with high Programmed Cell Death Ligand 1 (PD-L1) expression. Treatment with immune checkpoint inhibitors targeting PD-L1 was effective. This case highlights the clinical importance of considering accessory breast cancer as a distinct entity and demonstrates the potential utility of immunotherapy in such cases.

## Introduction

Aberrant breast tissue is defined as the ectopic glandular tissue near the primary breast, typically lacking a well-developed ductal system for secretion. It originates from the residual mammary tissue along the embryologic mammary ridge, commonly referred to as the 'milk line,' which extends bilaterally from the anterior axillary folds to the medial aspects of the thighs and crosses the inguinal region. The axilla is the most common site for aberrant breast tissue [1]. The incidence of accessory breast tissue varies among populations, with reported rates ranging from approximately 0.2% to 0.6%. It is more frequently observed in females and tends to be identified during puberty, pregnancy, or lactation due to hormonal changes. While reports from Asia are more prevalent, comprehensive and detailed data on regional differences in incidence remain limited [1-8]. Despite its low incidence, numerous case reports describing accessory breast cancer have been published [2-11]. Notably, nearly 80% of accessory breast cancers occur in the axillary region, corresponding to the most common site of accessory breast tissue [1].

While several cases of accessory breast cancer have been reported, synchronous primary cancers in the breast and ipsilateral accessory breast remain exceedingly rare. To our knowledge, no cases have been reported in which these synchronous tumors exhibit differing histopathological subtypes.

We, therefore, present a unique case of synchronous ipsilateral breast cancer and accessory breast cancer, each with a distinct subtype. The tumor in the left breast, located in the lower inner quadrant, exhibited a luminal-type phenotype. It was immunohistochemically positive for the estrogen and progesterone receptors (ER and PgR), negative for human epidermal growth factor receptor 2 (HER2) (score 0), and demonstrated a low Ki-67 labeling index of 3.2%. In contrast, the accessory breast tumor in the axilla displayed a triple-negative phenotype, with no expression of ER, PgR, or HER2 (score 0), and a high Ki-67 labeling index of 72.3%.

Triple-negative breast cancer (TNBC) is defined by the absence of ER, PgR, and HER2 expression. TNBC represents a particularly aggressive subtype of breast cancer, often associated with a poorer prognosis and limited treatment options compared to hormone receptor-positive or HER2-positive breast cancers. In recent years, the expression of programmed death-ligand 1 (PD-L1) on tumor cells or tumor-infiltrating immune cells has emerged as an important biomarker predicting the potential effectiveness of immune checkpoint inhibitors, such as pembrolizumab. Understanding these biological characteristics is crucial for optimizing treatment strategies, especially in rare and complex cases like the present one involving synchronous tumors with distinct histopathological profiles.

We discuss the clinical implications in light of the existing literature, highlighting the challenges in diagnosis and treatment of such rare and synchronous malignancies.

## Case presentation

**Figure 1:**
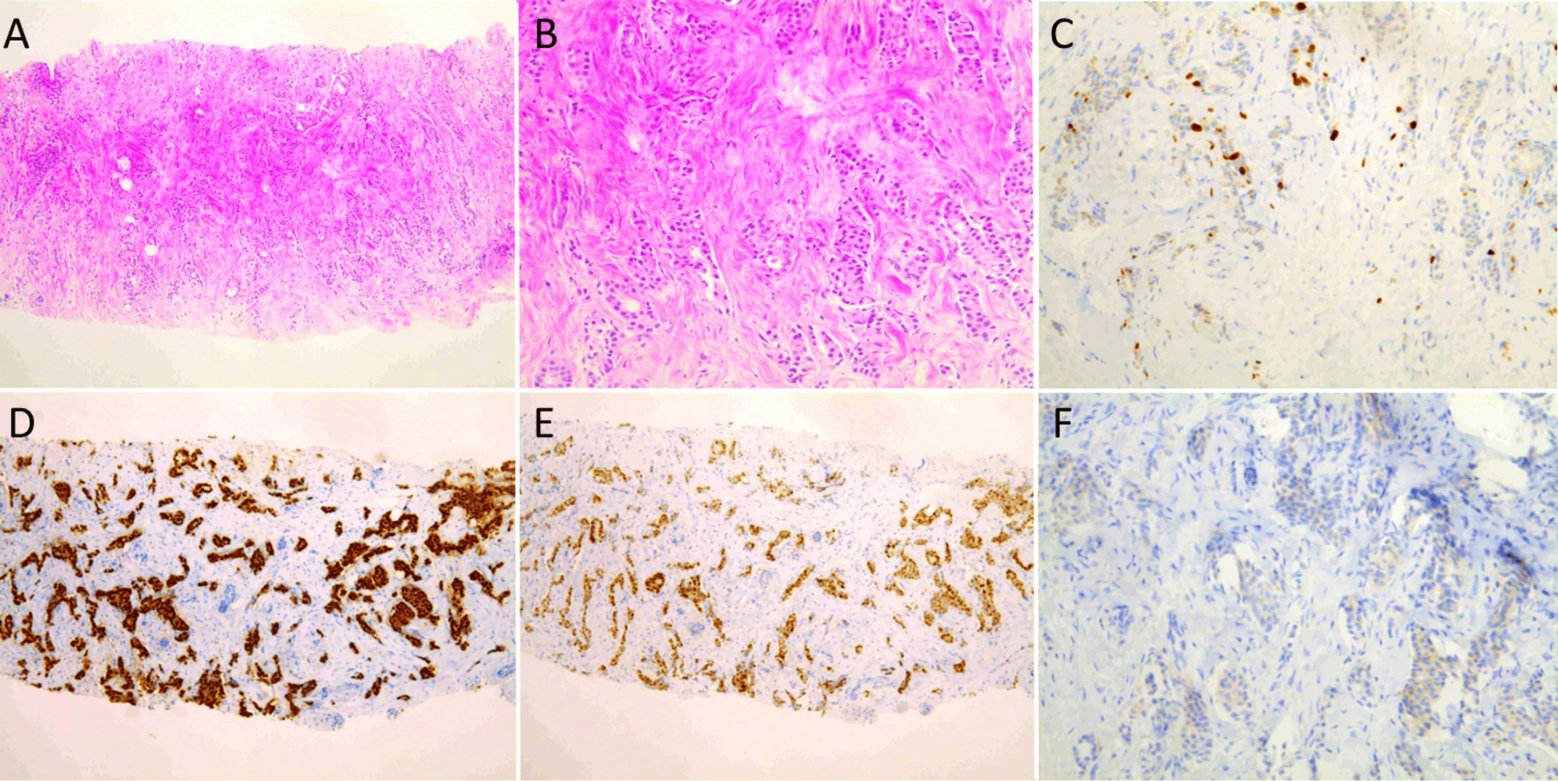
Microscopic appearance of the breast tumor at the time of the initial presentation at the previous hospital (one year and 10 months prior to referral to our institution) (A, B) Hematoxylin and eosin (H&E) staining of the core needle biopsy specimen showing invasive ductal carcinoma (low- and high-power views). Atypical epithelial cells with mild nuclear pleomorphism and no significant increase in mitotic figures infiltrating the fibroadipose tissue were observed. Normal mammary gland structures were absent in the specimen. (C) Immunohistochemistry (IHC) showing low proliferative activity, with a Ki-67 labeling index of 3.2%. (D) Positive staining for estrogen receptor (ER), Allred score TS8 (PS5+IS3). (E) Positive staining for progesterone receptor (PgR), Allred score TS6 (PS4+IS2). (F) Human epidermal growth factor receptor 2 (HER2) staining by IHC, scored as 0 (negative). Since no normal breast tissue was available for comparison within the specimen, typical immunohistochemical profiles of healthy breast tissue reported in the literature were used for reference. TS, Total Score; PS, Proportion Score; IS, Intensity Score

A 70-year-old female patient was referred to our hospital for continued treatment of breast cancer and a left axillary tumor. The breast tumor, located in the lower inner quadrant of the left breast, was immunohistochemically positive for ER and PgR), and negative for HER2 (score 0) (Figure 1).

At the previous hospital, the patient had undergone phased systemic therapy, including combination chemotherapy with anthracyclines for three months, bevacizumab and paclitaxel for 11 months, eribulin monotherapy for three months, and combination hormonal therapy with fulvestrant and abemaciclib for three months. As a result, the breast tumor had completely disappeared. However, the axillary tumor exhibited continuous progression through all lines of therapy with no evidence of treatment response. Given the persistent enlargement of the axillary tumor, the patient was referred to our hospital for cancer genome profiling and further treatment.

**Figure 2:**
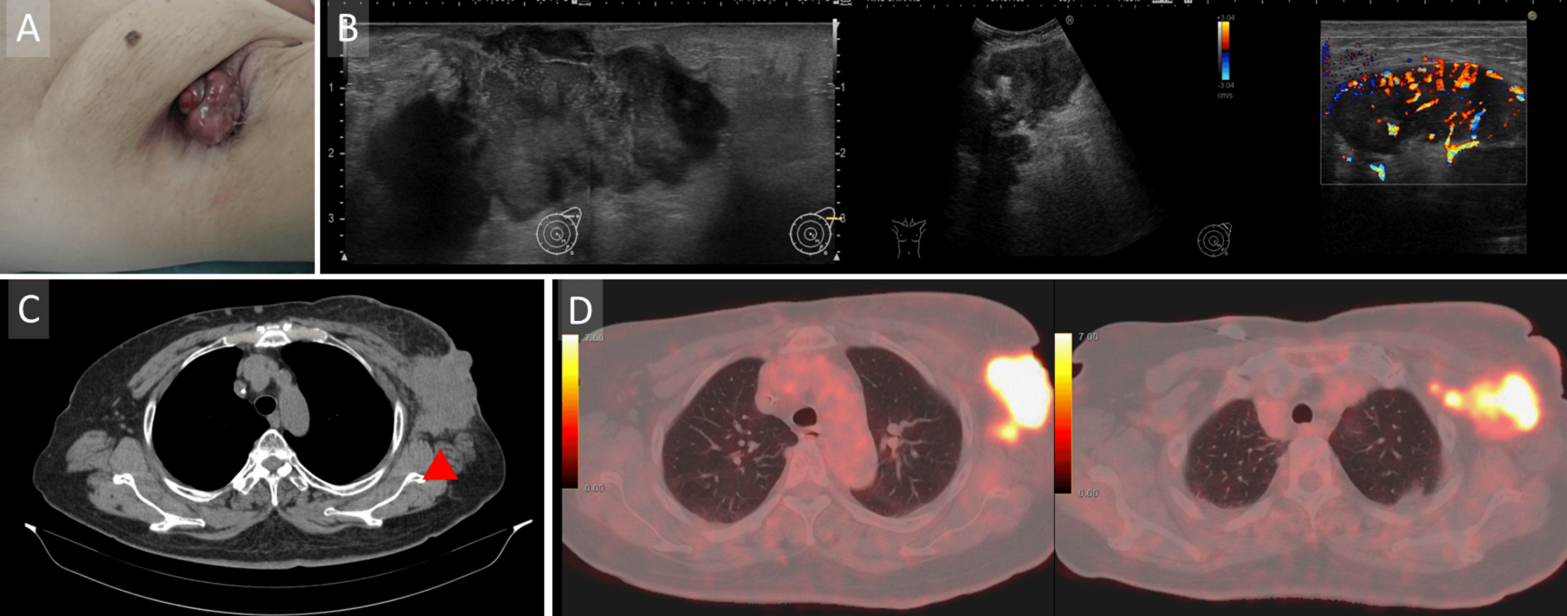
Preoperative examination (A) Clinical picture of the left axillary tumor, showing the tumor protruding and accompanied by erosion. (B) Ultrasound findings showing the ill-circumscribed, irregular-shaped, and heterogenous hypoechoic huge mass in the left axilla, and lymph nodes with vascular Doppler signaling. (C) CT findings showing the bulky mass exposed to the skin of the left axilla. (D) Fluorodeoxyglucose Positron Emission Tomography/Computed Tomography (FDG PET-CT) demonstrated FDG-accumulation in the left axillary lesion, from the cutaneous region to the axillary lymph nodes.

On presentation, the axillary mass was exposed on the skin surface and associated with an exudate and foul-smelling fluid (Figure 2A).

No lesions were detected in the breast on any imaging modality, presumably due to a favorable response to prior chemotherapy. The axillary lesion exhibited heterogeneous internal echoes and prominent Doppler signals (Figure 2B). Due to its large size and irregular shape, elasticity evaluation was difficult, and the posterior margin was poorly visualized on ultrasonography. The lesion appeared to arise from tissue beneath the left axillary skin, suggestive of accessory breast tissue. The patient underwent contrast-enhanced computed tomography (CT) of the chest, which revealed a large mass in the left axilla along with swollen axillary lymph nodes (Figure 2C). Fluorodeoxyglucose-positron emission tomography (FDG-PET) showed FDG accumulation in the left axillary lesion, extending from the cutaneous region to the axillary lymph nodes. No uptake was observed in any other lesions, including the breast, ruling out distant metastases (Figure 2D).

The patient had undergone two pregnancies and deliveries, with no history of excessive alcohol consumption or smoking. Aside from mild hypertension, her medical history was unremarkable. She had no history of asthma and there was no evidence of significant allergic conditions.

We initially suspected that the axillary region might not have lymph node metastasis from the primary tumor, so a needle biopsy of the axillary mass was performed. Histopathological examination showed that no breast glandular tissue was present in the specimen, as the glands were replaced by malignant cells. The mass was ultimately diagnosed as accessory breast cancer, as it was located at the axillary skin and was thought to originate from the subcutaneous tissue.

**Figure 3:**
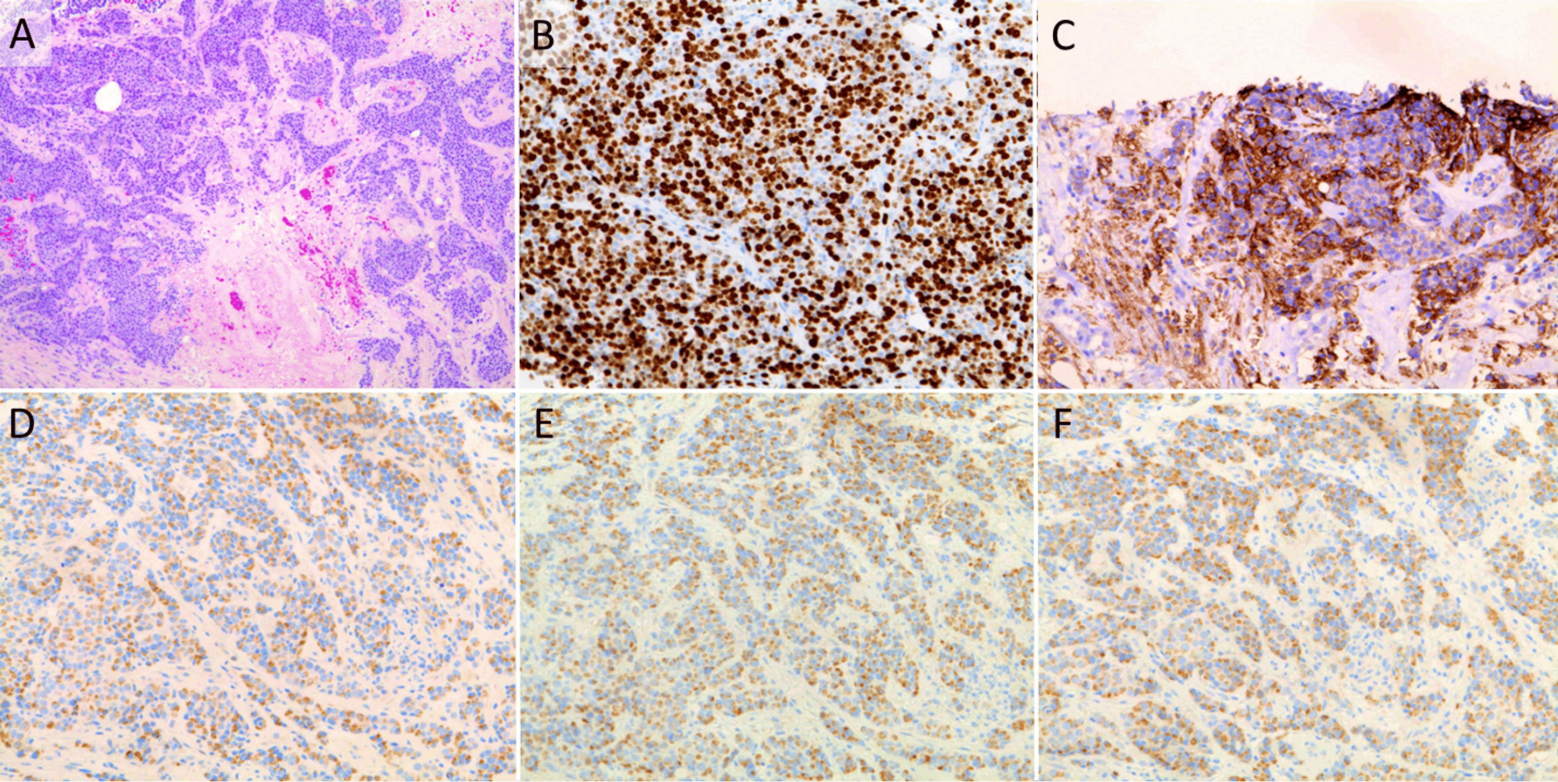
Microscopic appearance of the left axillary tumor at the time of initial assessment at our institution, with the specimen submitted for cancer genome profiling (CGP) (A) Hematoxylin and eosin (H&E) staining showing atypical epithelial cells with pleomorphic nuclei and frequent mitoses, infiltrating the surrounding fibroadipose tissue. No lymphoid tissue is identified. The tumor was classified as scirrhous-type invasive ductal carcinoma arising from accessory breast tissue. No normal mammary gland structures (such as ducts or lobules) are observed in the specimen, confirming the absence of normal breast tissue. Immunohistochemical findings of the tumor cells: (B) Ki-67 index of 72.3%. (C) Programmed Cell Death Protein 1 (PD-L1) expression, Combined Positive Score (CPS) of 27.3 in the Dako 22C3 immunohistochemistry assay. (D) Negative for estrogen receptor (ER). (E) Negative for progesterone receptor (PgR). (F) Negative for HER2, score 0.

Immunohistochemically, the tumor cells were negative for both ER and PgR, and HER2 was scored 0. The tumor was classified as triple-negative. Furthermore, high levels of PD-L1 expression were observed, with a combined positive score (CPS) of 27.3, as determined by the Dako 22C3 pharmDx immunohistochemistry assay (Agilent Technologies, Santa Clara, CA, USA) (Figure 3).

Genomic testing for breast cancer genes (BRCA) was performed to assess the potential benefit of the poly adenosine diphosphate ribose polymerase (PARP) inhibitor olaparib. However, no pathogenic BRCA variants were detected. Simultaneously, cancer genome profiling was conducted, revealing a high tumor mutation burden (TMB) of 153 mutations per megabase (Muts/Mb), suggesting potential efficacy with pembrolizumab. Sequence analysis also identified other pathogenic variants, including PIK3CA and KRAS (Table 1).

**Table 1:** Summary of the key findings from the cancer genomic profiling In addition to a high TMB, pathogenic variants in PIK3CA and KRAS were detected. All gene alterations follow standard Human Genome Variation Society (HGVS) protein-level nomenclature. Asterisks (*) indicate stop codons.

Gene	Alteration (*stop codon)	Variant allele frequency (%VAF)	Pathogenicity
PIK3CA	H1047R	19.2	Pathogenic
KRAS	G12V	2.3	Pathogenic
NF1	E106*	26.9	Likely pathogenic
ARID1A	E1683Q	20.9	Uncertain significance
TP53	R280T	0.2	Pathogenic
	E271Q	0.12	Likely pathogenic
	I255M	0.23	Likely pathogenic
ATRX	R907*	0.06	Likely pathogenic
BRCA1	E1765Q	0.17	Uncertain significance
Other findings			
Tumor mutational burden (TMB)			153 Muts/Mb
Microsatellite status (MS)			Stable

**Figure 4:**
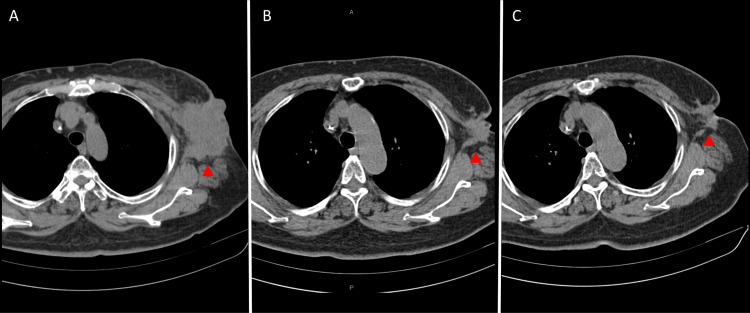
Time series of CT findings of the chest, showing the axillary tumor (▲) and its reduction in size over time following the initiation of immunotherapy-based combination therapy (A) At the first visit, when immunotherapy-based combination therapy was initiated. (B) Six months after the start of treatment, showing partial response. (C) One year later, with continued monitoring and ongoing treatment.

Based on these findings, combination chemotherapy with pembrolizumab, carboplatin, and gemcitabine was initiated. The treatment proved extremely effective, and the patient's condition improved remarkably (Figure 4).

Following the initiation of immunotherapy-based combination therapy, partial response was observed within six months, with continued monitoring over the next year. At the most recent follow-up, the treatment is ongoing, with further evaluations planned to assess the long-term efficacy and any potential recurrence.

## Discussion

Accessory breast cancer is a rare condition, accounting for only 0.2% to 0.6% of all breast cancers [1-8]. Selected cases identified through a PubMed search using the keyword "accessory breast cancer" with a publication date up to April 2025 are summarized in Table 2 [2-11].

**Table 2:** Studies involving patients with accessory breast carcinomas and their characteristics TN, triple negative; HR, hormone receptor; HER2, human epidermal growth factor receptor 2; ER, estrogen receptor; PgR, progesterone receptor

	Age	Gender	Location	Subtype
Zhang et al., 2020 [2]	48	Female	Right axilla	Luminal type
Miles et al., 2017 [3]	58	Female	Abdominal breast tissue	Luminal type
Yamamura et al., 2012 [4]	61	Male	Left axilla	Luminal type
Li et al., 2022 [5]	67	Male	Right axilla	TN
Zhang et al., 2015 [6]	45	Female	Left axilla	ER+ 9 cases; PgR+ 7cases; HER2+ 7cases
	48	Female	Right axilla	
	30	Female	Right axilla	
	27	Female	Left axilla	
	42	Female	Left axilla	
	38	Female	Right axilla	
	35	Female	Left axilla	
	48	Female	Left axilla	
	28	Female	Right axilla	
	46	Female	Right axilla	
	32	Female	Right axilla	
Reteria et al., 2023 [7]	65	Female	Right axilla	HR+, HER2－
Eguchi et al., 2021 [8]	68	Female	Inframammary region	HR+, HER2－
Fridman-Elder et al., 2021 [9]	68	Female	Right axilla	HR+, HER2－
Ji et al. 2023 [10]	76	Male	Left axilla	HR-, HER2: primary lesion－ and metastatic lesion (lung ) +
Yumoto et al., 2023 [11]	60	Female	Left axilla	ER+, PgR-, HER2－
Present case	70	Female	Left axilla	TN

Upon reviewing these reports, accessory breast cancer does not appear to be as rare as previously assumed. In our experience, such cases are encountered approximately once every year. Furthermore, considering that not all cases are likely to be reported in the literature, the true incidence may be higher than currently estimated. However, to our knowledge, there have been no previous reports describing the synchronous occurrence of a primary breast cancer within the mammary gland and an accessory breast cancer in the axilla, as observed in the present case. This suggests that the current case represents an exceptionally rare presentation.

The accessory breast is typically present at birth, most often located in the axilla, and is influenced by estrogen and progesterone [12]. In the present case, the cancer originating from the axillary accessory breast was diagnosed as TNBC, which appeared to be unresponsive to hormonal stimulation.

Breast cancer is a heterogeneous disease, and its subtypes are classified based on hormone receptor status, HER2 expression, and histological grade. TNBC, which lacks expression of ER, PgR, and HER2, is more likely to harbor stromal-infiltrating immune cells compared to luminal breast cancers [13]. The immune system plays a critical role in both the progression and control of breast cancer. Notably, TNBCs have been shown to be more responsive to immune checkpoint blockade than other subtypes, owing to their higher immunogenicity, greater infiltration by tumor-infiltrating lymphocytes (TILs), and elevated levels of PD-L1. In response to early immune activation, tumor cells and immune cells upregulate immune checkpoint molecules, and immunosuppressive metabolic pathways are concurrently activated [13-16]. TMB has also been correlated with the response to immune checkpoint blockade. TMB, defined as the total number of somatic mutations per coding area of the tumor genome, reflects the number of non-synonymous coding mutations within the tumor exome [17]. Tumors with a high mutational load may generate numerous neoantigens, potentially enhancing T-cell reactivity. In various malignancies, a TMB exceeding 10 mutations per megabase has been associated with improved clinical outcomes, leading to the inclusion of pembrolizumab as a treatment option for such patients. Moreover, pembrolizumab was approved in Japan in 2022 as a (neo)adjuvant therapy for high-risk, early-stage TNBC, based on promising clinical results [13,14,18]. In the present case, immunohistochemistry of the axillary tumor demonstrated elevated PD-L1 expression, and multigene panel testing revealed a high TMB, both of which supported the potential efficacy of pembrolizumab, and indeed, the patient showed a favorable response. 

The concept of hypermutated TMB, defined as ≥10 mutations per megabase (mut/Mb), is crucial in understanding the potential of immune checkpoint inhibitors like pembrolizumab. Although high TMB is not common in breast cancer compared to other malignancies, metastatic tumors tend to exhibit a higher prevalence of hypermutated TMB (8.4% in metastatic versus 2.9% in primary tumors). Specifically, TNBC has the highest median TMB among all breast cancer subtypes, though the frequency of hypermutated tumors is consistent across these subtypes. A study analyzing data from 3966 breast tumors reported that approximately 5% of all breast cancers had a TMB ≥10 mut/Mb. Notably, metastatic invasive lobular carcinoma has the highest frequency of hypermutated TMB. In patients with metastatic TNBC (mTNBC), high TMB is associated with improved progression-free survival (PFS) and overall survival (OS) when treated with immune checkpoint inhibitors, independent of clinical factors and PD-L1 status. This benefit was not seen in mTNBC patients treated with chemotherapy alone, underscoring the importance of TMB in predicting response to immune checkpoint inhibitor therapy [19].

Furthermore, in the present case, the PD-L1 CPS was 27.3, which is considered high. Given that high TMB has been associated with improved clinical outcomes with immune checkpoint inhibitors, and that a CPS ≥10 is similarly linked to better responses to pembrolizumab in TNBC, both biomarkers suggested a strong potential for therapeutic benefit. Clinical trials have demonstrated that patients with elevated PD-L1 expression are more likely to respond favorably to pembrolizumab-containing regimens. Thus, in addition to the high TMB, the high PD-L1 CPS further supported the selection of pembrolizumab for this patient and likely contributed to the favorable treatment outcome [17,20].

In addition, the cancer genome profiling results revealed mutations in PIK3CA and KRAS, suggesting an activation of the Ras/Mitogen-Activated Protein Kinase (RAS/MAPK) pathway. Although these mutations are not directly linked to an increased TMB, they may contribute to genomic instability, potentially supporting the observed high TMB status in this case. Furthermore, an activation of the RAS/MAPK pathway may play a role in treatment resistance. While these findings did not directly influence the initial treatment strategy, they highlight the potential utility of targeted therapies, such as PI3K, AKT, or MEK inhibitors, in the event of disease progression or recurrence [21]. Although routine reassessment of genomic alterations is not currently feasible under the healthcare system in Japan, careful clinical monitoring and consideration of additional genomic testing at the time of progression may guide future treatment decisions.

Genomic profiling tests in cancer patients may enable the selection of effective therapies that are not achievable through organ-specific approaches [22]. Given the accumulation of driver alterations during cancer progression, substantial heterogeneity has been recognized even within the same cancer type. As such, cancer genomic profiling has become an essential tool for determining the most appropriate treatment strategies, particularly for advanced cancers or those lacking established standard therapies [23]. The clinical utility of cancer genome sequencing is increasingly recognized, and it is anticipated that this approach will soon become more widely available to patients with earlier-stage cancers, including in Japan.

In the present case, the pathological diagnosis of PD-L1 positivity by immunohistochemistry and the indication for pembrolizumab based on the high TMB findings from genomic profiling were concordant. Combination chemotherapy with pembrolizumab proved effective, and the overall treatment strategy was considered successful. Initially, the treatment regimen was selected based on the clinical context in Japan, where the combination of pembrolizumab with chemotherapy is a limited approved and reimbursed option for PD-L1-positive TNBC. Following the results of cancer genome profiling, the possibility of switching to pembrolizumab monotherapy emerged as an alternative therapeutic strategy. However, the decision to transition to monotherapy will be carefully considered based on the patient’s clinical course and treatment response. 

## Conclusions

This case represents an exceptionally rare instance of synchronous malignancy involving both the breast and axillary accessory breast tissue. The clinical course underscores the diagnostic challenges posed by accessory breast cancer and highlights the importance of recognizing its potential coexistence with primary breast tumors. The favorable response to combined immunotherapy and chemotherapy suggests that such personalized treatment strategies may be effective even in atypical presentations.

Regarding the role of HER2 in immunotherapy, it remains a controversial and evolving topic. HER2-positive tumors are often responsive to HER2-targeted therapies, such as trastuzumab, and there is increasing interest in combining these therapies with immunotherapy to enhance treatment outcomes. However, the precise role of HER2 in the immunotherapy response, particularly in the context of immune checkpoint inhibitors, is still under investigation. In contrast, HER2-negative tumors may benefit from immune checkpoint inhibitors, but the relationship between HER2 expression and immune response remains unclear. Further research is needed to elucidate the implications of HER2 status in the context of immunotherapy, as its potential as a predictive biomarker is not yet fully established. Although rare, these cases may offer valuable insights into tumor biology, immune responsiveness, and the evolving landscape of precision oncology.
